# Nm23/nucleoside diphosphate kinase-A as a potent prognostic marker in invasive pancreatic ductal carcinoma identified by proteomic analysis of laser micro-dissected formalin-fixed paraffin-embedded tissue

**DOI:** 10.1186/1559-0275-9-8

**Published:** 2012-06-27

**Authors:** Tatsuyuki Takadate, Tohru Onogawa, Kiyonaga Fujii, Fuyuhiko Motoi, Sayaka Mikami, Tetsuya Fukuda, Makoto Kihara, Takashi Suzuki, Taro Takemura, Takashi Minowa, Nobutaka Hanagata, Kengo Kinoshita, Takanori Morikawa, Keiichi Shirasaki, Toshiki Rikiyama, Yu Katayose, Shinichi Egawa, Toshihide Nishimura, Michiaki Unno

**Affiliations:** 1 Division of Gastroenterological Surgery, Department of Surgery, Tohoku University Graduate School of Medicine, 1-1 Seiryo-machi, Aoba-ku, Sendai, 980-8574, Miyagi, Japan; 2 Department of Structural Biology, Hokkaido University Graduate School of Pharmaceutical Sciences, Sapporo, Japan; 3 Biosys Technologies, Incorporated, Tokyo, Japan; 4 Medical ProteoScope Company, Limited, Kanagawa, Japan; 5 Department of Pathology and Histotechnology, Tohoku University Graduate School of Medicine, Sendai, Japan; 6 Nanotechnology Innovation Station, National Institute for Materials Science, Tsukuba, Japan; 7 Department of Applied Information Sciences, Tohoku University Graduate School of Information Science, Sendai, Japan; 8 Division of Integrated Surgery and Oncology, Tohoku University Graduate School of Medicine, Sendai, Japan; 9 Department of Surgery I, Tokyo Medical University, Tokyo, Japan

**Keywords:** Proteomics, Prognostic biomarker, Formalin-fixed paraffin-embedded (FFPE), Laser micro-dissection (LMD), Liquid chromatography-tandem mass spectrometry (LC-MS/MS), Nm23/Nucleoside Diphosphate Kinase A (NDPK-A)

## Abstract

**Background:**

Pancreatic cancer is among the most lethal malignancies worldwide. This study aimed to identify a novel prognostic biomarker, facilitating treatment selection, using mass spectrometry (MS)-based proteomic analysis with formalin-fixed paraffin-embedded (FFPE) tissue.

**Results:**

The two groups with poor prognosis (n = 4) and with better prognosis (n = 4) had been carefully chosen among 96 resected cases of pancreatic cancer during 1998 to 2007 in Tohoku University Hospital. Although those 2 groups had adjusted background (UICC-Stage IIB, Grade2, R0, gemcitabine adjuvant), there was a significant difference in postoperative mean survival time (poor 21.0 months, better 58.1 months, *P* = 0.0067). Cancerous epithelial cells collected from FFPE tissue sections by laser micro-dissection (LMD) were processed for liquid chromatography-tandem mass spectrometry (LC-MS/MS). In total, 1099 unique proteins were identified and 6 proteins showed different expressions in the 2 groups by semi-quantitative comparison. Among these 6 proteins, we focused on Nm23/Nucleoside Diphosphate Kinase A (NDPK-A) and immunohistochemically confirmed its expression in the cohort of 96 cases. Kaplan-Meier analysis showed high Nm23/NDPK-A expression to correlate with significantly worse overall survival (*P* = 0.0103). Moreover, in the multivariate Cox regression model, Nm23/NDPK-A over-expression remained an independent predictor of poor survival with a hazard ratio of 1.97 (95% CI 1.16-3.56, *P* = 0.0110).

**Conclusions:**

We identified 6 candidate prognostic markers for postoperative pancreatic cancer using FFPE tissues and immunohistochemically demonstrated high Nm23/NDPK-A expression to be a useful prognostic marker for pancreatic cancer.

## Background

Pancreatic cancer has the worst prognosis of any major malignancy, with a 5-year survival rate of less than 5% after diagnosis [1]. It is the fifth leading cause of cancer death and the incidence of pancreatic cancer is rising in Japan [2,3]. The majority of exocrine pancreatic cancers are invasive ductal carcinomas. Radical surgical resection is the only chance for cure, but only 10% to 20% of patients are candidates for curative resection, [2] and even if curative resection is performed, the postoperative 5-year survival rate is only 15% to 25% because of high recurrence rates [4,5]. In an effort to explain improved survival rates, several studies have analyzed determinants of long-term survival in postoperative pancreatic cancer patients [6-15]. However, the prognosis of pancreatic cancer patients has improved little in the past 20 years [2]. Thus, there is an urgent need to identify novel molecular targets for early diagnosis and selecting optimal treatments.

In recent decades, many exploratory studies have aimed to discover biomarkers for early detection, optimal treatment selection and predicting therapeutic outcomes, with the goal of improving prognosis [16]. Proteomics has become a major tool for the discovery of diagnostic and prognostic cancer biomarkers, but pancreatic cancer analysis is challenging due to histological heterogeneity within the tumor. However, laser micro-dissection (LMD) provides an ideal method of extracting tumor specimens and isolating targeted cells with defined morphologies. Formalin-fixed paraffin-embedded (FFPE) tissues, long collected and stored in hospitals worldwide, represent an enormous untapped information resource concerning disease progression as well as drug responses and toxicity. FFPE tissue is particularly advantageous for discovering biomarkers because of the wealth of accompanying clinical data [17,18].

We performed retrospective proteomics by liquid chromatography–tandem mass spectrometry (LC-MS/MS) using FFPE tissues from resected invasive pancreatic ductal carcinomas and semi-quantitative group comparison. To identify novel prognostic biomarkers, we selected 2 groups with poor prognosis and better prognosis. We focused on differentially expressed proteins between the 2 groups and immunohistochemically confirmed the candidate proteins in a cohort of 96 cases of invasive pancreatic ductal carcinoma.

## Methods

### Patient characteristics and FFPE tissue samples

**Figure 1:**
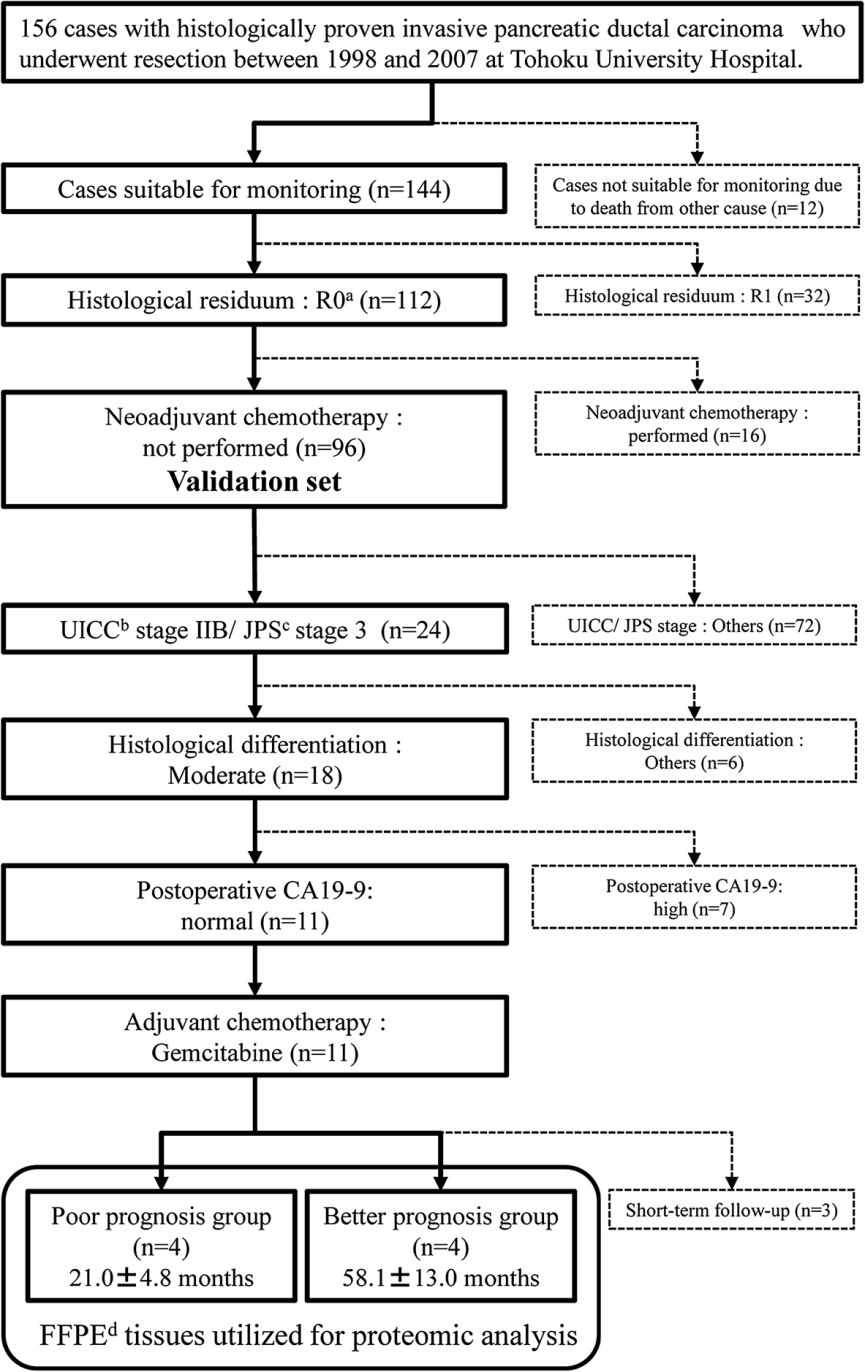
** Patient selection for proteomic analysis.**^a^ Histological residuum R0 shows microscopically negative margins. ^b^ Unio Internationalis Contra Cancrum/Union for International Cancer Control. UICC stage IIB: Cancer has spread to nearby lymph nodes and may also have spread to adjacent tissues and organs. ^c^ Japan Pancreas Society. JPS stage 3: Cancer has not extended into the portal vein, extra-pancreatic nerve plexus, or other organs in UICC stage IIB. ^d^ Formalin-Fixed Paraffin-Embedded

The study subjects were selected from among 156 histologically diagnosed invasive pancreatic ductal carcinoma cases undergoing pancreatectomy during the January 1998 through December 2007 period at our hospital. We selected 96 cases suitable for monitoring, with microscopic complete resection (R0), and no neoadjuvant chemotherapy (Figure 1). To identify novel biomarkers, we standardized known prognostic factors: 1) pathological stage, [6-8,10-12,14] 2) histological differentiation, [10,12,14] 3) postoperative carbohydrate antigen 19–9 (CA19-9) level, [19] and 4) adjuvant chemotherapy [9,10,13,15]. We excluded 3 cases because the observation period was too short, leaving 8 cases divided into 2 groups, poor prognosis (n = 4) and better prognosis (n = 4), for shotgun proteomic analysis (Figure 1). There was a significant difference in postoperative mean survival time (poor 21.0 ± 4.8 months, better 58.1 ± 13.0 months, *P* = 0.0067). First, we performed shotgun proteomic analysis using FFPE tissues from these 8 invasive pancreatic ductal carcinomas. FFPE tissues were utilized for this semi-quantitative proteomic study with approval from the Ethics Committee of Tohoku University (2006–119), and informed consent was obtained from individual patients.

### LMD and protein extraction

Tumor tissues were fixed in 4% paraformaldehyde and routinely processed for paraffin sectioning. Cancerous lesions were identified on serial sections stained with hematoxylin and eosin. For shotgun proteomic analysis, 10-μm sections prepared from the same tissue block were attached to DIRECTOR™ slides (Expression Pathology, MD, USA), de-paraffinized three times with xylene for 5 min, rehydrated with graded ethanol solutions and distilled water, and then stained with hematoxylin. Stained, uncovered slides were air-dried and approximately 30,000 cancerous cells (8 mm^2^) were collected into the cap of a 0.2 mL PCR tube using Leica LMD6000 (Leica Microsystems GmbH, Germany). Peptides were extracted using a Liquid Tissue™ MS Protein Prep kit (Expression Pathology) [20] according to the manufacturer’s instructions. Briefly, the cellular material, suspended in the Liquid Tissue buffer, was incubated at 95 °C for 90 min, then cooled on ice for 3 min. Trypsin was added (approximately 15–18 U) followed by incubation at 37 °C overnight. Dithiothreitol was added to a final concentration of 10 mM, and the samples were heated for 5 min at 95 °C. The Liquid Tissue digestate was stored at −20 °C until analysis.

### LC-MS/MS shotgun analysis

Exploratory LC-MS/MS analysis of a digested sample was performed using reversed phase (RP)-LC interfaced with an LTQ-Orbitrap hybrid mass spectrometer (Thermo Fisher Scientific, Germany) using a nanoelectrospray device (AMR, Tokyo, Japan), as previously reported [21]. The RP-LC system (Paradigm MS4B, Michrom BioResources, CA, USA) consisted of a peptide Cap-Trap cartridge (2.0 × 0.5 mm inside diameter) and an analytical column (L-column Micro, 150 × 0.2 mm L-C18, 3 μm, 12 nm, Chemical Evaluation Research Institute, Tokyo, Japan) fitted with an emitter tip (FortisTip, OmniSeparo-TJ, Hyogo, Japan). Peptide-mixture samples processed from FFPE tissues were loaded onto the trap cartridge and washed with mobile phase A (98% H_2_O with 2% acetonitrile and 0.1% formic acid) for concentration and desalting. Subsequently, the samples were eluted over 70 min from the analytical column via the trap cartridge using a linear gradient of 5–40% mobile phase B (10% H_2_O with 90% acetonitrile and 0.1% formic acid) at a flow-rate of 1 μL/min. General MS conditions were as follows: electrospray voltage, 3.0 kV, no sheath and auxiliary gas flow; ion transfer tube temperature, 200 °C; collision energy, 35%; ion selection threshold, 1000 counts for MS/MS. MS/MS was performed on the top 3 ions in each MS scan using the dynamic exclusion principle, i.e. temporary (180 s) placement of a mass on an exclusion list after its MS/MS spectrum has been acquired.

### Data analysis and protein identification

All MS/MS spectral data were searched against *Homo sapiens* entries in the Swiss-Prot database (Release 57.13, 20,349 entries) using MASCOT software (version 2.1.1, Matrix Science, UK). The peptide mass tolerance was 20 ppm, fragment mass tolerance 0.8 Da, and trypsin specificity was applied with a maximum of 2 missed cleavages. For variable peptide modifications, methionine oxidation, and N-formylation, including formyl (K), formyl (R), and formyl (N-terminus), were taken into account. To estimate the false positive rate for protein identification, a decoy database was created by reversing the protein sequences in the original database. Based on search results for the decoy database, the estimated false positive rate of peptide matches was 4.0% under *P* < 0.05 for protein score threshold conditions. Results were obtained from triplicate LC-MS/MS runs for each sample.

### Spectral counting analysis of identified proteins

To compare protein expression across all tissue samples from the results of shotgun analysis, I used the label-free spectral counting method [18]. The number of peptide spectra with high confidence (Mascot ion score, *P* < 0.05) served as the spectral count value. All proteins with at least one peptide spectrum in a single LC-MS/MS analysis were considered for protein quantification using spectral counting. The averaged values based on triplicate analyses were estimated as the spectral count values of each protein for individual tissue samples, taking into consideration statistical significance.

Fold changes in expressed proteins on a base-2 logarithmic scale were calculated using the protein ratio from spectral counting (Rsc) [22]. Rsc > 1 or < −1 corresponds to fold changes > 2 or < 0.5. Differences in relative abundances of identified proteins were also assessed by spectral index (SpI), allowing group comparison [23]. SpI values ranged from −1 to +1, and those close to 0 indicated near-equal relative peptide abundance in the 2 groups. Candidate proteins of the 2 groups chosen based on Rsc > 1 or < −1 and SpI > 0.75 or < -0.75, showed statistical significance at *P* < 0.05 by non-parametric *G*-test [24].

### Immunohistochemistry

Immunohistochemistry was performed using FFPE tissue sections stained with the horseradish peroxidase EnVision + System (DAKO, CA, USA). Antigen retrieval was performed by heating the sections in 10 mmol/L citrate buffer (pH 6.0) for 5 minutes. Mouse monoclonal nm23/nucleoside diphosphate kinase-A (Nm23/NDPK-A) antibody, clone37.6 (Abcam, MA, USA), was used as the primary antibody at a 1:100 dilution. A breast cancer tissue section served as the positive control [25]. For negative controls, mouse IgG2a isotype control (R&D Systems, MN, USA) was used as the primary antibody. The sections were lightly counterstained with hematoxylin. After completely reviewing all slides of immunostained sections for each carcinoma, three of the authors (T.T., T.O. and T.S.) independently and blindly classified carcinoma cases into 2 groups: those in which the percentage of carcinoma cells positive for Nm23/NDPK-A exceeded 10% were the positive group, while the negative group comprised those with fewer than 10% positive cells, as in previous studies [26,27].

### Statistical analysis

The χ^*2*^-test was used to compare categorical variables. In univariate analysis, survival rates were calculated by the Kaplan-Meier method and compared using the log-rank test. In multivariate analysis, independent prognostic factors were determined by the Cox proportional hazards model. *P* < 0.05 was considered statistically significant. JMP software version 9.0 was used for all analyses. Analyses were performed using only available data; missing information was assumed to be non-informative.

## Results

**Figure 2:**
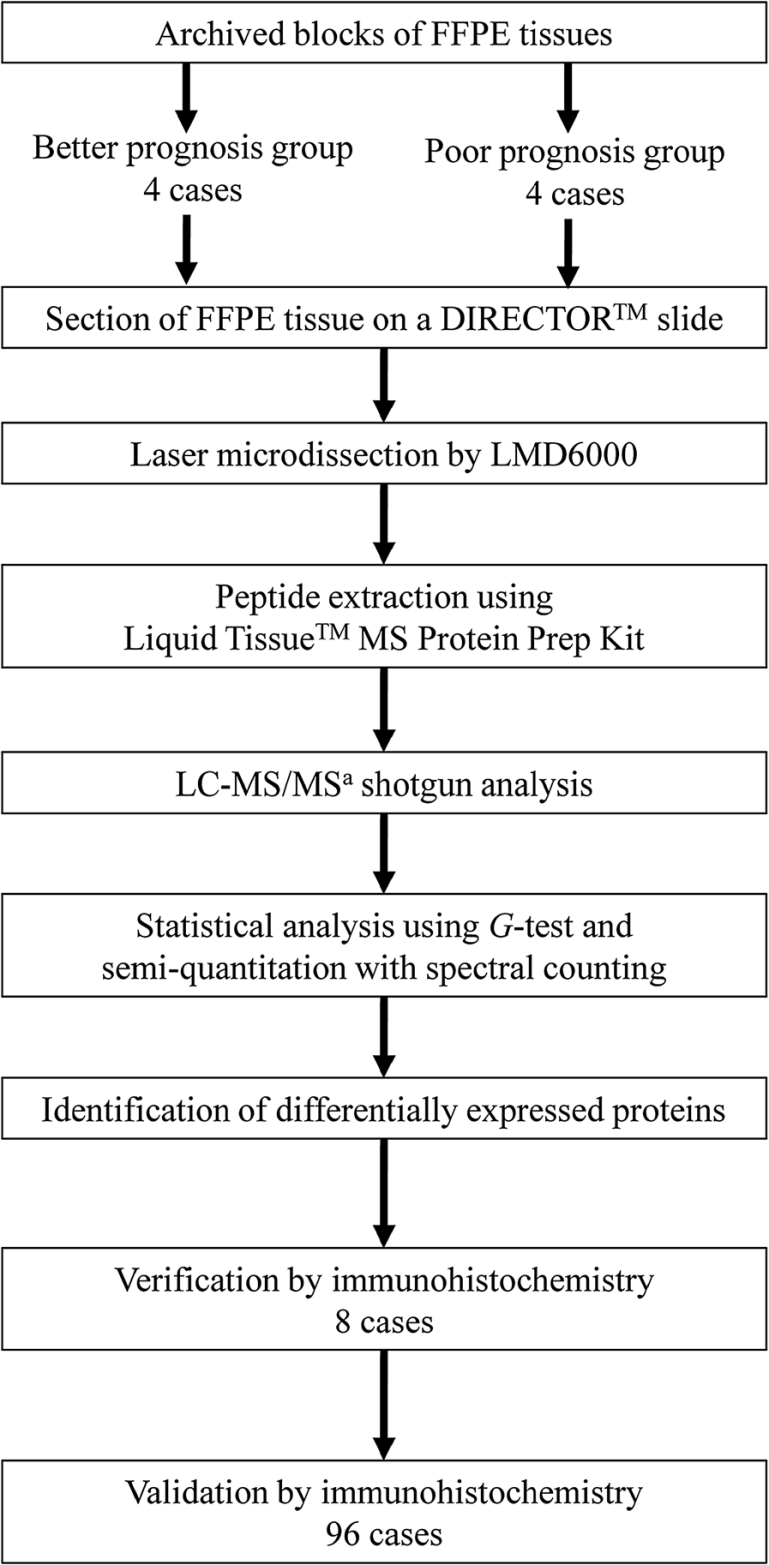
** Strategy for shotgun proteomic analysis and immunohistochemical confirmation.**^a^ Liquid chromatography-tandem mass spectrometry

The present study aimed to investigate molecular profiles of proteins relevant to pancreatic cancer and to confirm these proteins to be potential prognostic biomarkers. The workflow is illustrated in Figure 2.

### Patient characteristics

First, we performed shotgun proteomic analysis using FFPE tissues from 8 invasive pancreatic ductal carcinomas. The better and poor prognostic groups each provided 4 independent primary tumor tissues. The 2 groups did not differ in age, gender, tumor location, pathological staging, lymph node metastasis, tumor differentiation, postoperative CA19-9, or adjuvant chemotherapy (Table 1).

**Table 1:** Patients characteristics in the poor and better prognostic group

**Variable**	**Category**	**Poor prognostic group (n = 4)**	**Better prognostic group (n = 4)**	***P*value**
Postoperative survival months	Mean ± SD^a^	21.0 ± 4.80	58.1 ± 13.0	0.0067^b^
Age	Mean ± SD	66.75 ± 10.66	63.25 ± 12.95	0.6909^c^
Gender	Male	3(75)	3(75)	
	Female	1(25)	1(25)	
Location	Head	4(100)	4(100)	
T	T3	4(100)	4(100)	
N	N1	4(100)	4(100)	
M	M0	4(100)	4(100)	
UICC stage	IIB	4(100)	4(100)	
JPS stage	3	4(100)	4(100)	
Differentiation	Moderate	4(100)	4(100)	
Residuum	R0	4(100)	4(100)	
Postoperative CA19-9	<37 U/ml	4(100)	4(100)	
Adjuvant chemotherapy	Gemcitabine	4(100)	4(100)	

^a^ Standard deviation.

^b^ The log-rank test was used.

^c^ The *t*-test was used.

### Protein identification and semi-quantitative comparison

In shotgun proteomic analysis, 845 proteins in the better prognostic group, 924 in the poor prognostic group and 1099 proteins in total were identified ( Additional file 1: Table S1). The identified proteins were semi-quantitatively compared using spectral counting analysis. We identified that ADP-ribosylation factor 4 (ARF4), Collagen alpha-3(VI) chain (CO6A3), DNA-binding protein A (DBPA), Malate dehydrogenase, cytoplasmic (MDHC), Nucleoside diphosphate kinase A (NDKA) and Probable transcription factor PML (PML) were differentially expressed in the 2 groups, including 4 and 2 over-expressed proteins in the poor and better prognostic groups, respectively (Table 2).

**Table 2:** Proteins identified differentially expressed between better and poor prognostic groups

**Accession number**	**Entry name**	**Protein name**						
			**Better prognostic group**	**Poor prognostic group**	**Fold change (Rsc**^**a**^**)**	**Spectral index (SpI)**	***G*score**	***P*-value**
P15531	NDKA_HUMAN	Nucleoside diphosphate kinase A	1	6	1.74	0.82	3.88	0.049
P16989	DBPA_HUMAN	DNA-binding protein A	2	10	1.85	0.79	5.89	0.015
P18085	ARF4_HUMAN	ADP-ribosylation factor 4	5	25	2.13	0.79	15.1	<0.01
P12111	CO6A3_HUMAN	Collagen alpha-3(VI) chain	6	31	2.21	0.76	19.2	<0.01
P29590	PML_HUMAN	Probable transcription factor PML	10	1	−2.27	−0.89	7.85	<0.01
P40925	MDHC_HUMAN	Malate dehydrogenase, cytoplasmic	7	0	−2.67	−1	8.81	<0.01

^a^ protein ratio from spectral counting.

### Nm23/NDPK-A expression in invasive pancreatic ductal carcinoma

**Figure 3:**
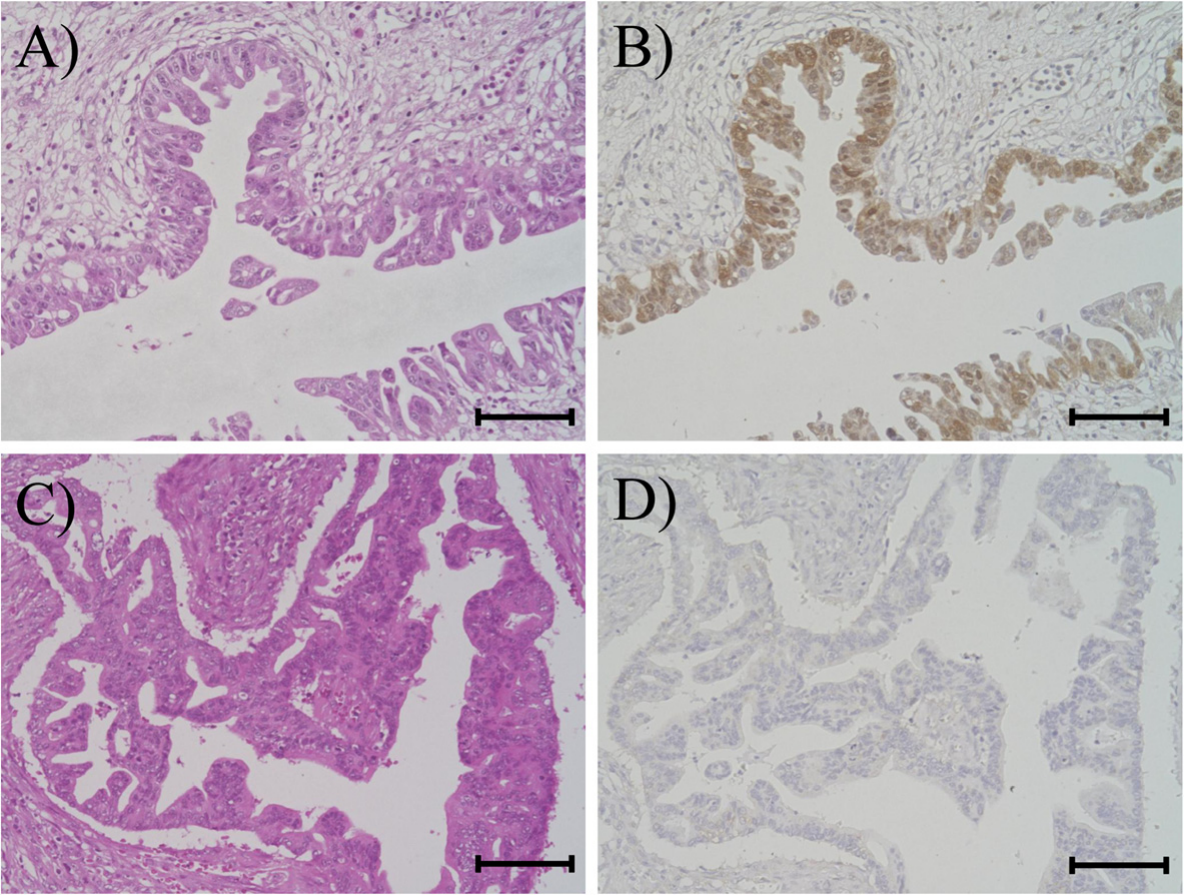
** Immunohistochemistry for Nm23/NDPK-A in invasive pancreatic ductal carcinoma.A**), **B**) hematoxylin-eosin staining, **C**) Nm23/NDPK-A immunoreactivity was detected in the cytoplasm of carcinoma cells. **D**) No Nm23/NDPK-A immunoreactivity was detected in carcinoma cells. Scale bar = 100 μm, original magnification, ×200

To confirm expressions of the 6 candidate proteins in tissue sections, we immunohistochemically analyzed the 8 cases with shotgun proteomic data. Among the 6 candidate proteins, we focused on NDKA (also called Nm23/NDPK-A), because the immunohistochemical results were most compatible with those of spectral counting analysis. Nm23/NDPK-A has previously been described as being associated with prognosis in non-pancreatic cancers. Nm23/NDPK-A was over-expressed in the poor prognostic group on semi-quantitative comparison. The immunostaining results for Nm23/NDPK-A are shown in Figure 3. As with semi-quantitative analysis, 3 of 4 cases were negative in the better prognostic group, while all 4 cases in the poor prognostic group were positive for Nm23/NDPK-A, which was detected in the carcinoma cell cytoplasm.

### Relationships between Nm23/NDPK-A expression and clinicopathological features in 96 invasive pancreatic ductal carcinoma patients

To confirm the association between Nm23/NDPK-A and the prognosis of invasive pancreatic ductal carcinoma, we immunohistochemically analyzed a 96-case cohort (62 men, 34 women; mean age, 64 years). Baseline characteristics are presented in Table 3. Median survival of patients in this cohort was 19.1 months and 20.4% were alive at 5 years. Nm23/NDPK-A was expressed in the carcinoma cell cytoplasm in 73 of the 96 (76%) tumor specimens. Nm23/NDPK-A expression status was compared across survival-associated clinical parameters (Table 3). Nm23/NDPK-A staining patterns did not differ by age, gender, tumor location, histological differentiation, pathological stage, lymph node metastasis, National Comprehensive Cancer Network (NCCN) resectability status, pre- and postoperative CA19-9 levels, or adjuvant chemotherapy. It was suggested that Nm23/NDPK-A was a potentially independent prognostic factors of pancreatic cancer. Thus, we had investigated the correlation between the expression of Nm23/NDPK-A and prognosis.

**Table 3:** Patient characteristics as stratified by Nm23/NDPK-A status

**Variable**	**Category**	**All patients (n = 96)**	**Nm23/NDPK-A positive (n = 73)**	**Nm23/NDPK-A negative (n = 23)**	***P*value**
Age	< 65	45 (47%)	32 (44%)	13 (57%)	0.2877
	≥ 65	51 (53%)	41 (56%)	10 (43%)	
Gender	Male	62 (65%)	45 (62%)	17 (74%)	0.2833
	Female	34 (35%)	28 (38%)	6 (26%)	
Location	Head	73 (76%)	56 (77%)	17 (74%)	0.7839
	Body-Tail	23 (24%)	17 (23%)	6 (26%)	
Histological differentiation^a^	Well	8 (8%)	6 (8%)	2 (9%)	0.9838
	Moderate + Poor	77 (80%)	58 (80%)	19 (82%)	
Tumor stage	T1 + T2	10 (10%)	7 (10%)	3 (13%)	0.6363
	T3 + T4	86 (90%)	66 (90%)	20 (87%)	
Nodal stage	N0	33 (34%)	25 (34%)	8 (35%)	0.9624
	N1	63 (66%)	48 (66%)	15 (65%)	
NCCN^b^ criteria	Resectable	50 (52%)	38 (52%)	12 (52%)	0.9920
	Borderline rsectable	46 (48%)	35 (48%)	11 (48%)	
Preoperative CA19-9	< 37 U/mL	25 (26%)	17 (23%)	8 (35%)	0.2733
	≥ 37 U/mL	71 (74%)	56 (77%)	15 (65%)	
Postoperative CA19-9	< 37 U/mL	57 (59%)	41 (56%)	16 (70%)	0.2538
	≥ 37 U/mL	39 (41%)	32 (44%)	7 (30%)	
Adjuvant chemotherapy	Gemcitabine	58 (60%)	42 (58%)	16 (70%)	0.3035
	Others^c^	38 (40%)	31 (42%)	7 (30%)	

^a^ Other histological classifications including papillary, mucinous, adenosquamous and anaplastic carcinoma in 11 cases.

^b^ National Comprehensive Cancer Network.

^c^ Others include 5FU and surgery alone.

The χ^*2*^-test was used to compare categorical variables. None of the categories was significantly associated with Nm23/NDPK-A status.

### Survival and the expression of Nm23/NDPK-A

**Figure 4:**
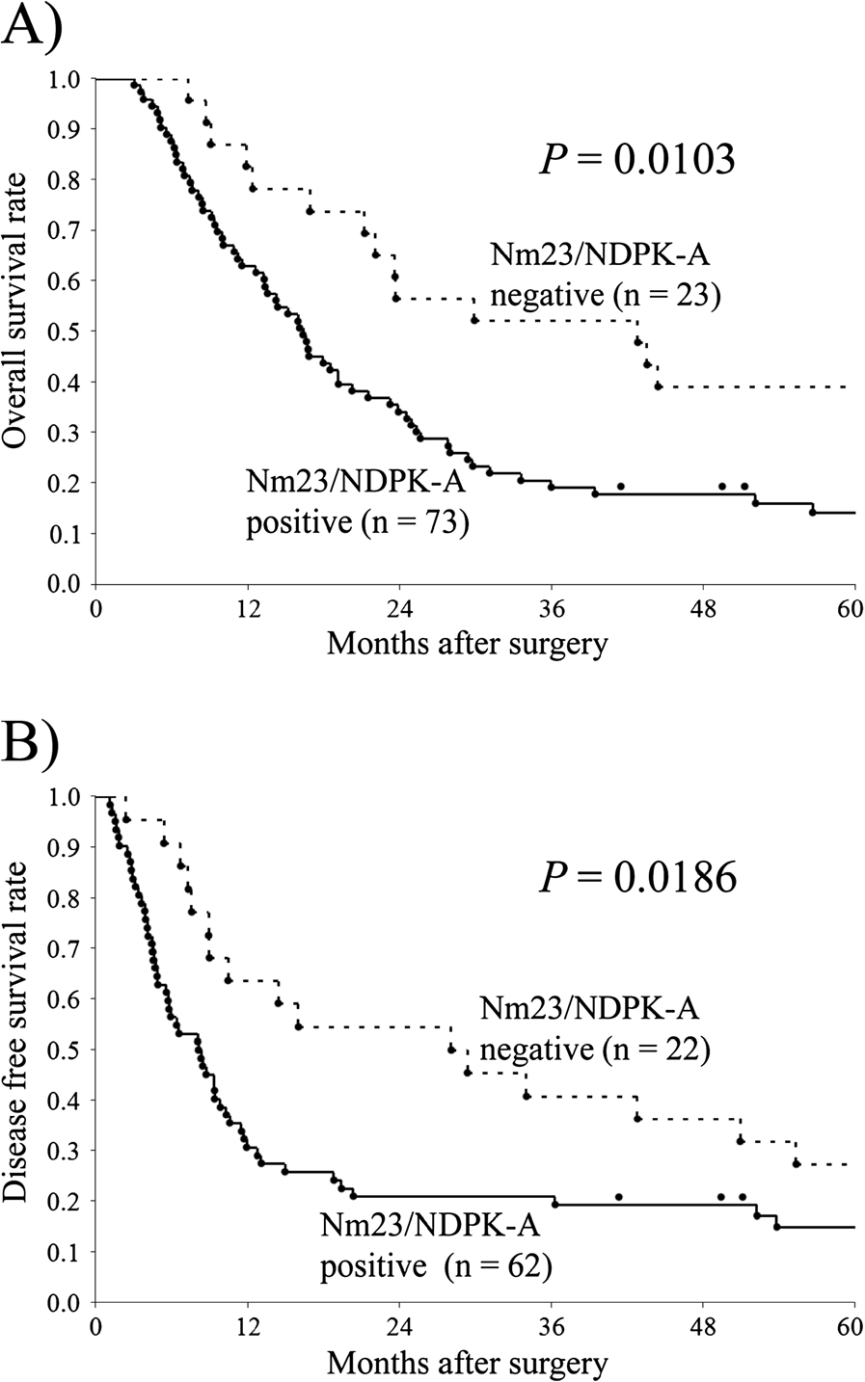
** Survival analysis for Nm23/NDPK-A immunoreactivity by the Kaplan-Meier method.A**) Nm23/NDPK-A positivity was significantly associated with poor overall survival (log-rank test, *P* = 0.0103). **B**) Nm23/NDPK-A positivity was significantly associated with poor disease free survival (log-rank test, *P* = 0.0186). The day of recurrence was unclear in 11 cases

Kaplan-Meier analysis revealed patients with carcinoma cells expressing Nm23/NDPK-A to have significantly worse overall survival (OS) and disease free survival (DFS) than those without Nm23/NDPK-A (Figure 4, OS; *P* = 0.0103, DFS; *P* = 0.0186). In univariate analysis, lymph node metastasis (*P* = 0.0039), NCCN criteria (*P* = 0.0339), postoperative CA19-9 level (*P* = 0.0082), and Nm23/NDPK-A expression (*P* = 0.0103) were significantly associated with worse OS (Table 4). With the multivariate Cox regression model, lymph node metastasis (Hazard Ratio [HR] 2.20, 95% Confidence Interval [CI] 1.33-3.80, *P* = 0.0020), postoperative CA19-9 level (HR 1.92, 95% CI 1.21-3.04, *P* = 0.0062), and Nm23/NDPK-A expression (HR 1.97, 95% CI 1.16-3.56, *P* = 0.0110) remained independent factors predicting poor survival (Table 4).

**Table 4:** Univariate and Multivariate analyses of clinicopathological factors influencing overall survival

**Variable**	**Category**	**No. of patients**	***P*value by log-rank test**	**HR**^**a**^	**95% CI**^**b**^	***P*value by Cox proportional hazards**
Gender	Male	62	0.1288			
	Female	34				
Age	< 65	45	0.8967			
	≥ 65	51				
Location	Head	73	0.8589			
	Body-Tail	23				
Histological differentiation	Well	8	0.2069			
	Moderate + Poor	77				
Tumor stage	T1 + T2	10	0.1996			
	T3 + T4	86				
Nodal stage	N0	63	0.0039	2.20	1.33-3.80	0.0020
	N1	33				
NCCN criteria	Resectable	50	0.0339	1.40	0.90-2.21	0.1419
	Borderline resectable	46				
Preoperative CA19-9	< 37 U/mL	25	0.3444			
	≥ 37 U/mL	71				
Postoperative CA19-9	< 37 U/mL	57	0.0082	1.92	1.21-3.04	0.0062
	≥ 37 U/mL	39				
Adjuvant chemotherapy	Gemcitabine	58	0.4201			
	Others	38				
Nm23/NDPK-A status	Negative	23	0.0103	1.97	1.16-3.56	0.0110
	Positive	73				

^a^ Hazard ratio.

^b^ Confidence Interval.

Univariate analysis was performed using the log-rank test. Multivariate analysis was performed using the Cox proportional hazard model for variables that were significant on univariate analysis.

## Discussion

Recently, efforts have been made to identify novel biomarker proteins by gene microarray and proteomics methods. Pancreatic cancer is aggressive and has an extremely poor prognosis due to delayed diagnosis, early metastasis, and resistance to most cytotoxic agents. Thus, it is very important to find new diagnostic, prognostic and therapeutic biomarkers [16]. We explored novel independent prognostic biomarkers of pancreatic cancer by shotgun proteomics using LC-MS/MS, with clinically-documented micro-dissected FFPE tissues, spectral counting for semi-quantification and immunohistochemical confirmation.

FFPE, a routine process providing easily-stored archived tissues, is well-defined pathologically. Extensive FFPE tissue archives have been collected and stored at room temperature for decades in hospitals worldwide. FFPE tissue contains associated clinical and experimental information representing an extremely valuable, untapped reservoir of protein biomarkers [18,20]. For decades, however, FFPE tissue has been used only for light microscopic diagnosis and staging of tumors [28]. A methodological limitation is that, due to various combinations of intra- and inter-molecular covalent cross-linking between proteins by formaldehyde, proteins are preserved but rendered insoluble [28-30]. Recent developments in extraction methodologies have finally made analysis of FFPE tissue by MS possible, providing access to this vast and clinically important sample set for biomarker discovery [18,20,31-33]. By spectral counting for semi-quantification, we identified 6 proteins differentially expressed in the 2 groups, including 4 and 2 over-expressed proteins in the poor and better prognostic groups, respectively. Though we confirmed all 6 candidate proteins, we focused on Nm23/NDPK-A because its immunohistochemical results were most compatible with those of spectral counting analysis. Nm23/NDPK-A has previously been described as being associated with prognosis in non-pancreatic cancers.

The *NM23* gene family was initially identified as putative metastasis suppressors based on reduced expression in certain highly metastatic cell lines and tumors [34,35]. In humans, 10 genes belong to the *NM23* gene family (also known as *NME* genes). The two most abundantly expressed are *NM23-H1* and *NM23-H2* encoding the A and B subunits, respectively, of NDPK [36]. Nm23/NDPK-A has several biochemical functions [37]: 1) nucleoside diphosphate kinase, 2) phosphotransferase and histidine protein kinase, 3) 3’-5’-exonuclease, and 4) regulation of GTP-binding proteins. Inverse correlations between *NM23-H1* expression and high tumor metastatic potential in several tumor types, e.g. hepatocellular carcinoma, melanomas, breast cancer, ovarian cancer and gastric cancer, have been demonstrated [38-41] In other human carcinomas, such as colon cancer, pancreatic ductal carcinoma, neuroblastoma and non-Hodgkin’s lymphoma, high mRNA and protein levels of *NM23-H1* have been detected in aggressive tumors [25,42-47]. Andolfo *et al* reported that *NM23-H1* functioned as an inhibitor of tumor invasion *in vitro*, but had the opposite function *in vivo*[27]*.* Although the mechanisms underlying these differences are presently unknown, the action of Nm23/NDPK-A might be altered or regulated in different ways in various organs, under the influence of other genes and proteins.

Only a few studies have examined Nm23/NDPK-A expression in pancreatic cancer. Nakamori *et al* reported strong immunoreactivity for Nm23/NDPK-A and -B to be associated with lymph node metastasis and poor prognosis in 47 pancreatic cancer cases [25,44]. We immunohistochemically analyzed Nm23/NDPK-A in 96 cases, a larger sample size than that of Nakamori *et al*. Our findings also indicated higher Nm23/NDPK-A expression levels to be associated with poor prognosis, but there was no significant correlation with lymph node metastasis. Furthermore, in our study, Nm23/NDPK-A expression was an independent prognostic factor for OS on multivariate analysis. Ohshio *et al* found no significant correlation between Nm23/NDPK-A expression and prognosis of resected cases [42]. This discrepancy in results between our study and that of Ohshio *et al* might reflect differences in sample case backgrounds. Nakamori *et al* and our group examined only resected pancreatic cancer samples whereas Ohshio *et al* investigated samples from both resected pancreatic cancers and metastases.

Although further study is needed to determine the precise role of Nm23/NDPK-A in malignant behavior of cells, Nm23/NDPK-A expression is potentially useful for assessing prognosis or selecting treatments in pancreatic cancer patients. Positivity for Nm23/NDPK-A in resected specimens might serve as an index providing information useful to physicians for the administration of adjuvant therapy and intensive follow-up of cancer patients likely to suffer a recurrence. By combining the expression of Nm23/NDPK-A, lymph node matastasis and postoperative CA19-9 level that were independent prognostic factors in this study, it might be possible to know the prognosis and make treatment more accurately. Herein, Nm23/NDPK-A expression was confirmed immunohistochemically, though Okabe-Kado *et al* reported that the serum Nm23/NDPK-A level may contribute to predicting prognosis of neuroblastoma patients [48]. It would be interesting to examine whether serum Nm23/NDPK-A levels are associated with poor prognosis of postoperative pancreatic cancer patients. Identification of Nm23/NDPK-A by immunocytochemistry using endoscopic ultrasound-guided fine needle aspiration or brushing cytology specimens might be also anticipated to be useful for diagnosis and selecting optimal treatments for preoperative patients. Moreover, if the precise role of Nm23/NDPK-A in malignant behavior of cells is elucidated, Nm23/NDPK-A might be therapeutic target.

## Conclusions

We performed a proteomic analysis to identify novel prognostic biomarkers for postoperative pancreatic cancer using FFPE tissues. We semi-quantitatively compared expressed proteins between poor and better prognostic groups. The results were immunohistochemically confirmed. A high level of Nm23/NDPK-A expression correlated with poor OS and DFS. Measurement of Nm23/NDPK-A expression is potentially useful for obtaining prognostic and treatment information for pancreatic cancer patients. MS-based proteomic analysis with FFPE tissue offers new opportunities to identify biomarkers and therapeutic targets using archival samples with their corresponding pathological and clinical records.

## Abbreviations

ARF4: ADP-ribosylation factor 4; CA19-9: Carbohydrate antigen 19–9; CI: Confidence interval; CO6A3: Collagen alpha-3(VI) chain; DBPA: DNA-binding protein A; DFS: Disease free survival; FFPE: Formalin-fixed paraffin-embedded; HR: Hazard ratio; JPS: Japan pancreas society; LC: Liquid chromatography; LMD: Laser micro dissection; MDHC: Malate dehydrogenase: cytoplasmic; MS: Mass spectrometry; MS/MS: Tandem mass spectrometry; NDKA: Nucleoside diphosphate kinase A; Nm23/NDPK-A: Nm23/nucleoside diphosphate kinase-A; NCCN: National comprehensive cancer network; OS: Overall survival; PML: Probable transcription factor PML; RP: Reverse phase; Rsc: Protein ratio from spectral counting; SpI: Spectral index; UICC: Unio internationalis contra cancrum/Union for International Cancer Control.

## Competing interests

This work was supported in part by Grants-in-Aid for young scientists (A) (19689028) and scientific research (B) (22390254), the Nanotechnology Network Japan Program and the Network Medicine Global-COE Program from the Ministry of Education, Culture, Sports, Science and Technology of Japan (MEXT).

The authors have no potential conflicts of interest to disclose.

## Authors’ contributions

TT was involved in study concept and design, acquisition of data, analysis and interpretation of data, drafting of the manuscript, critical revision of the manuscript for important intellectual content, statistical analysis, and obtaining funding. TO was involved in study concept and design, acquisition of data, analysis and interpretation of data, critical revision of the manuscript for important intellectual content, statistical analysis, obtaining funding, and study supervision. KF was involved in acquisition of data, analysis and interpretation of data, technical support, and approved the manuscript. FM was involved in study concept and design, acquisition of data, analysis and interpretation of data, critical revision of the manuscript for important intellectual content, statistical analysis, obtaining funding, and study supervision. SM was involved in acquisition of data, analysis and interpretation of data, technical support, and approved the manuscript. TF was involved in acquisition of data, analysis and interpretation of data, technical support, and critical revision of the manuscript for important intellectual content. MK was involved in acquisition of data, analysis and interpretation of data, technical support, and approved the manuscript. TS was involved in acquisition of data, analysis and interpretation of data, and approved the manuscript. T Takemura was involved in technical support, and approved the manuscript. T Minowa was involved in technical support, and approved the manuscript. NH was involved in technical support, and approved the manuscript. KK was involved in analysis and interpretation of data and approved the manuscript. T Morikawa was involved in study concept and design, critical revision of the manuscript for important intellectual content, and obtaining funding. KS was involved in technical support, and approved the manuscript. TR was involved in study concept and design, critical revision of the manuscript for important intellectual content, and obtaining funding. YK was involved in study concept and design, critical revision of the manuscript for important intellectual content, and obtaining funding. SE was involved in study concept and design, and critical revision of the manuscript for important intellectual content. TN was involved in study concept and design, analysis and interpretation of data, critical revision of the manuscript for important intellectual content, and study supervision. MU was involved in study concept and design, critical revision of the manuscript for important intellectual content, obtaining funding, and study supervision. All authors read and approved the final manuscript.

## Supplementary Material

Additional file 1: Table S1. **The list of all proteins identified in shotgun proteomic analysis.** (XLS 264 kb) Click here for file
